# Destabilization of Lysophosphatidic Acid Receptor 1 Reduces Cytokine Release and Protects Against Lung Injury

**DOI:** 10.1016/j.ebiom.2016.07.020

**Published:** 2016-07-18

**Authors:** Jing Zhao, Jianxin Wei, Su Dong, Rachel K. Bowser, Lina Zhang, Anastasia M. Jacko, Yutong Zhao

**Affiliations:** a Department of Medicine, University of Pittsburgh, School of Medicine, Acute Lung Injury Center of Excellence, Vascular Medical Institute, United States; b Department of Cell Biology, University of Pittsburgh, Pittsburgh, PA, United States; c Department of Anesthesia, First Hospital of Jilin University, Changchun, China; d Department of Critical Care Medicine, Xiangya Hospital, Central South University, Changsha, Hunan, China

**Keywords:** GPCR, Ubiquitination, Deubiquitination, TLR4/CD14, Lung injury

## Abstract

Lysophosphatidic acid receptor 1 (LPA1) is a druggable target for treating pulmonary inflammatory diseases. However, the molecular regulation of LPA1 stability, a factor that critically impacts its biological activity, remains largely unknown. Here we identify two enzymes that regulate the balance of LPA1 ubiquitination and deubiquitination. Ubiquitin E3 ligase Nedd4L targets LPA1 for its site specific ubiquitination and degradation in the lysosome. Nedd4L negatively regulates LPA-LPA1-mediated cytokine release. The stability of LPA1 is up-regulated by ubiquitin-specific protease 11 (USP11), which deubiquitinates LPA1 and enhances LPA1-mediated pro-inflammatory effects. LPA1 is associated with USP11 in quiescent cells, while LPA treatment triggers LPA1 dis-association with USP11 and in turn binding to Nedd4L. Knockdown or inhibition of USP11 reduces LPA1 stability, levels of LPA1, and LPA1-CD14 interaction complex; thereby diminishing both LPA- and LPS-induced inflammatory responses and lung injury in preclinical murine models. Thus, our findings identify an ubiquitin E3 ligase and a deubiquitinating enzyme responsible for regulation of LPA1 stability and biological activities. This study provides potential targets for the development of anti-inflammatory molecules to lessen lung injury.

## Highlights

• Nedd4L ubiquitinates LPA1 and diminishes LPA1 signaling.
• USP11 deubiquitinates and stabilizes LPA1, thus promotes LPA1- and LPS-mediated pro-inflammatory response.
• Inhibition of USP11 reduces LPS-induced acute lung injury.

Lysophosphatidic acid (LPA) and its receptor (LPA1) play a critical role in lung inflammation through triggering cytokine release and neutrophil influx to the lungs. Here, we show that LPA1 is ubiquitinated and degraded in the lysosome, and the process is mediated by the ubiquitin E3 ligase Nedd4L. Further, we reveal that a deubiquitination enzyme USP11 stabilizes LPA1 by targeting and deubiquitinating LPA1. To investigate whether destabilization of LPA1 diminishes lung injury, USP11 was inhibited or down-regulated prior to endotoxin challenge. Destabilization of LPA1 reduces cytokine release and lung inflammation in a preclinical murine model of lung injury.

## 1 Introduction

In the United States, approximately 200,000 people suffer annually from acute lung injury (ALI) and the acute respiratory distress syndrome (ARDS), with a high mortality rate ranging from 30 to 40% (Rubenfeld et al., 2005). Acute respiratory infection commonly elicits a cytokine storm, leading to epithelial and endothelial barrier disruption, edema, and often respiratory failure. Lipopolysaccharide (LPS), a bacterial endotoxin, induces a strong cytokine storm through its receptor complex composed of toll-like receptor 4 (TLR4) and CD14 (Lu et al., 2008, Ojaniemi et al., 2003, Park and Lee, 2013). In addition to binding to TLR4, CD14 associates with other cell surface proteins, such as surfactant protein C (Augusto et al., 2003), to modulate LPS-induced inflammatory responses. Our recent study revealed that CD14 interacts with the lysophosphatidic acid receptor 1 (LPA1), which belongs to G-protein-coupled receptors (GPCRs) (Zhao et al., 2011).

LPA1 is highly expressed in lung tissue and contributes to the pathogenesis of lung injury through binding to LPA (Zhao and Natarajan, 2013, Lin et al., 2010), a naturally occurring bioactive lipid, and its interaction with CD14 (Zhao et al., 2011). We have shown that LPA1-modulated signals are involved in release of chemotactic factor interleukin 8 (IL-8) (Saatian et al., 2006, Cummings et al., 2004, Zhao et al., 2005), neutrophil influx to the lungs (He et al., 2009), and airway epithelial hyperplasia (Funke et al., 2012). The protein level of LPA1 is increased in lung inflammatory disorders, including asthma (Georas et al., 2007), pulmonary fibrosis (Tager et al., 2008), and ALI (Zhao et al., 2011). LPS- or bleomycin-induced lung injury is diminished in LPA1-deficient mice (Zhao et al., 2011, Tager et al., 2008); therefore, LPA1 is a potential target for pharmaceutical treatment of lung injury. However, the molecular regulation of LPA1 has not been well studied.

The mono-ubiquitination-lysosome system degrades the majority of cell surface receptors including GPCRs (Marchese and Trejo, 2013, Marchese et al., 2003, Shenoy et al., 2008, Henry et al., 2012, Xiao and Shenoy, 2011). Three enzyme complexes (E1, E2, and E3) are involved in protein ubiquitination. E3 ubiquitin ligases facilitate the covalent attachment of ubiquitin to specific lysine residue(s) within target proteins. The E3 ubiquitin ligase for LPA1 has not been reported. Nedd4L, a member of HECT class of E3 ubiquitin ligase has been known to catalyze ubiquitination of cell surface and intracellular proteins, such as epithelial sodium channel (ENaC) (Kamynina et al., 2001), Smad2, and Smad3 (Gao et al., 2009). Here we demonstrate that the ubiquitin E3 ligase Nedd4L mediates LPA1 ubiquitination and lysosomal degradation, therefore limiting LPA1-modulated signaling.

Protein ubiquitination is reversible; removal of ubiquitin chains from target proteins is mediated by deubiquitinating enzymes. We have shown that ubiquitin-specific protease (USP) 14 modulates I-κB levels (Mialki et al., 2013). Little is known about the role of USPs in the regulation of GPCR stability. USP11, a ubiquitous protein in various human cells, has been shown to enhance TGFβ receptor (ALK5) stability (Al-Salihi et al., 2012) and regulate DNA repair (Wiltshire et al., 2010). The current study reveals that LPA1 is a substrate for USP11, and inhibition of USP11 mitigates lung injury through reduction of LPA1 levels and LPA1-CD14 pathway.

## 2 Materials and Methods

### 2.1 Cells and Reagents

Murine lung epithelial (MLE12) cells (from ATCC) were cultured with HITES medium containing 10% fetal bovine serum (FBS) and antibiotics at 37 °C in 5% CO_2_. Primary culture of human bronchial cells (HBEpCs) (from Lonza, Baltimore, MD) was conducted using medium supplemented with growth factors provided by Lonza. V5 antibody, mammalian expressional plasmid pcDNA3.1-His-V5-topo, and *Escherichia coli* Top10 competent cells were from Life technologies (Grand Island, NY). P-p38 MAPK, p38 MAPK, p-IκB, Nedd4L, HA tag, and ubiquitin antibodies were from Cell Signaling (Danvers, MA). LPA1 and LPA2 antibodies were from LifeSpan BioScience, Inc. (Seattle, WA). Cycloheximide (CHX, 3-[2-(3,5-Dimethyl-2-oxocyclohexyl)-2-hydroxyethyl]glutarimide), leupeptin (Acetyl-Leu-Leu-Arg-al), lipopolysaccharide (LPS), β-actin and myc tag antibodies were from Sigma (St. Louis, MO). MG-132 (Z-L-Leu-D-Leu-L-Leu-al) and bafilomycin A1 (C35H58O9) were from EMD Chemicals (Philadelphia, PA). Immunobilized protein A&G beads, control IgG, p-Erk1&2, Erk1&2, and USP11 antibodies were from Santa Cruz Biotechnology (Santa Cruz, CA). All materials in highest grades used in the experiments are commercially available.

### 2.2 Plasmid and shRNA Transfection

Human *LPA1*, human *NEDD4L*, or human *USP11* cDNA, and mutants were inserted into pCDNA3.1-V5-His-Topo vector, pCDNA3.1-HA vector, or pCDNA3.1-myc vector. All the primers were designed using Primer3 or QuickChange Primer Design Tool (Agilent Technologies Inc.) software. Over-expression of plasmids in MLE12 cells was performed using the Lonza nucleofector system. Over-expression of plasmids in HBEpCs was performed using FuGENE HD reagent (Promega, Madison, WI).

### 2.3 Preparation of Protein Extracts and Immunoblotting

After indicated treatments, cells were lysed in 1 × lysis buffer (Cell signaling). Equal amount of total protein were subjected to SDS-PAGE gel, transferred to nitrocellulose, and then immunoreacted with primary antibody, followed by secondary antibody.

### 2.4 Co-Immunoprecipitation

Equal amounts of protein were incubated with primary antibody for overnight at 4 °C, followed by adding protein A&G beads for additional 2 h in room temperature. The beads were precipitated by centrifugation at 1000*g* for 2 min, and then were rinsed with PBS for 3 times. Proteins on the beads were eluted by boiling in SDS sample buffer.

### 2.5 Immunostaining

MLE12 cells were cultured in glass-bottom dishes and fixed with 3.7% formaldehyde for 20 min. Permeabilization in 0.1% Triton-100 for 1 min was performed for determining localization of LPA1-V5, LPA1-myc, HA-Nedd4L, or USP11-V5. Cells were exposed to primary antibody, followed by incubation with fluorescence-labeled secondary antibody. Immunofluorescent cell imaging was performed using a Zeiss LSM 510 confocal microscope.

### 2.6 Reverse Transcription (RT) Realtime PCR

Cells were collected after indicated treatment, and then total RNA was extracted using Trizol reagent from Life Technologies. 1 μg of RNA was used for reverse transcription reaction to generate cDNA. Realtime PCR was performed using Bio-Rad Ssofast Evagreen supermix reagent with synthesized cDNA as template. PCR primers were designed for detecting human IL-8, IL-6, and mouse KC gene.

### 2.7 Animals

C57/BL6 mice (6–8/group) were given intratracheal (i.t.) LPS (2 mg/kg body weight) for 24 h. BAL fluid was collected for cytokine analysis using ELISA. Mouse *Usp11* shRNA was inserted into a pLVX-IRES vector (Clontech); Lenti-shUSP11 viral and control viral vectors were generated by using a lentivirus packaging system (Clontech). C57/BL6 mice were given i.t. Lenti-control or Lenti-USP11 shRNA (10^9^ plaque-forming units/mouse) for 7 days prior to i.t. inoculation with LPS (2 mg/kg weight) for 24 h. BAL fluid was collected for cytokine assays and lung tissues were fixed for hematoxylin and eosin (H&E) staining. To determine the effect of MX on lung inflammation, C57/BL6 were given i.t. MX (0.25 mg/kg body weight) prior to LPS challenge, and then BAL fluids and lung tissues were randomly and blindly analyzed as described above. All animal procedures in this study were performed in adherence with the National Institute of Health Guidelines on the use of Laboratory Animals and have been approved by the Institutional Animal Care and Use Committee of the University of Pittsburgh.

### 2.8 Statistical Analysis

All results were subjected to statistical analysis using Microsoft Excel or ANOVA, and, wherever appropriate, the data were analyzed by Student's *t*-test and expressed as means ± SD. Data were collected from at least three independent experiments, and *p* < 0.05 was considered significant.

## 3 Results

### 3.1 Site Specific Ubiquitination of LPA1 Promotes LPA1 Degradation

**Fig. 1:**
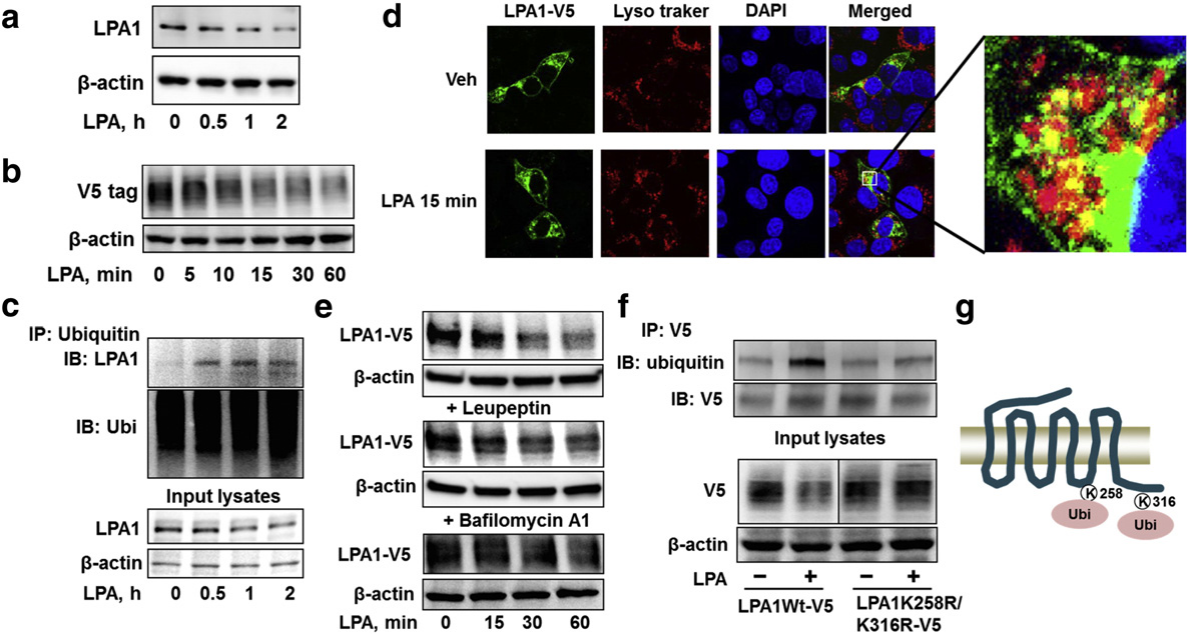
LPA1 lysosomal degradation is mediated by site specific ubiquitination. **a**. MLE12 cells were treated with LPA (5 μM) for 0–2 h, cell lysates were then analyzed by immunoblotting with LPA1 and β-actin antibodies. **b**. MLE12 cells were transfected with V5 tagged LPA1 (*LPA1-V5*) plasmid for 48 h, followed by LPA (5 μM, 0–60 min) treatment. Cell lysates were then analyzed by immunoblotting with V5 and β-actin antibodies. **c**. MLE12 cells were treated with LPA (5 μM) for 0–2 h. Cell lysates were then subjected to immunoprecipitation with an ubiquitin antibody, followed by LPA1 immunoblotting. Input lysates were analyzed by immunoblotting with LPA1 and β-actin antibodies. **d**. MLE12 cells grown on glass-bottom dishes were transfected with *LPA1-V5* plasmid for 48 h, followed by LPA (5 μM, 15 min) treatment. Cells were fixed and stained with V5, lyso tracker, and 4′,6′-diamidino-2-phenylindole (DAPI). LPA1-V5, green; lysosome, red; nuclei, blue. **e**. MLE12 cells were transfected with *Lpa1-V5* plasmid for 48 h, and then cells were treated with or without leupeptin (100 μM) or bafilomycin A1 (0.1 μM) for 1 h prior to LPA (5 μM, 0–60 min) treatment. Cell lysates were analyzed by immunoblotting with V5 and β-actin antibodies. **f**. MLE12 cells were transfected with *Lpa1-V5* or *Lpa1k258r&k316r-V5* plasmid for 48 h, and then cells were treated with LPA (5 μM, 1 h). Cell lysates were subjected to immunoprecipitation with a V5 antibody, followed by ubiquitin and V5 immunoblotting. Input lysates were analyzed by immunoblotting with V5 and β-actin antibodies. **g**. Scheme shows LPA1 is ubiquitinated on lysine 258 and 316 residues. Representative immunoblots were from at least three independent times.

Ligand-induced receptor degradation plays a negative feedback loop to attenuate membrane receptor signaling. To investigate the molecular regulation of LPA1 degradation, murine lung epithelial (MLE12) cells were treated with LPA, and protein levels of endogenous and over-expressed V5-tagged LPA1 (LPA1-V5) were examined by immunoblotting. As shown in Fig. 1a and b, LPA treatment reduces LPA1 levels in a time dependent manner. As ubiquitination is a recognized signal for receptor degradation, we examined the whether LPA1 is ubiquitinated and its role in LPA1 degradation. LPA1 ubiquitination was examined by immunoprecipitation with an ubiquitin antibody, followed by LPA1 immunoblotting. LPA treatment induced mono-ubiquitination of LPA1 in 30 min (Fig. 1c). To determine whether LPA1 is degraded in the lysosome, the localization of LPA1-V5 was determined by immunofluorescence staining. LPA1-V5 was co-localized with lysosome marker after LPA treatment (Fig. 1d). Further, inhibition of lysosome function by leupeptin or bafilomycin A1 impaired LPA-induced LPA1-V5 degradation (Fig. 1e), indicating that LPA1 degradation occurs in the lysosome. As ubiquitin mostly ligates to lysine residues (K) within the substrates, we attempted to identify which lysine residue is the ubiquitination site within LPA1. LPA1 contains two lysine residues (K258 and K316) in the intracellular domains. We generated plasmids coding signal lysine mutants (LPA1K258R or LPA1K316R) and double lysine mutant (LPA1K258R&K316R). LPA1K258R&K316R was resistant to LPA-induced ubiquitination and degradation (Fig. 1f), while LPA1K258R or LPA1K316R had no effect on LPA1 stability (supplemental Fig. 1), indicating that both the lysine residues are necessary for LPA1 ubiquitination and stability. Mono-ubiquitination contributes to internalization of cell surface receptors. However, LPA induced internalization of both LPA1-V5 and ubiquitination site mutants (supplemental Fig. 2). The data suggests that ubiquitination at K258R&K316 regulates LPA1 degradation (Fig. 1g), but it is not essential for LPA1 internalization.

### 3.2 Nedd4L is the Ubiquitin E3 Ligase for LPA1

**Fig. 2:**
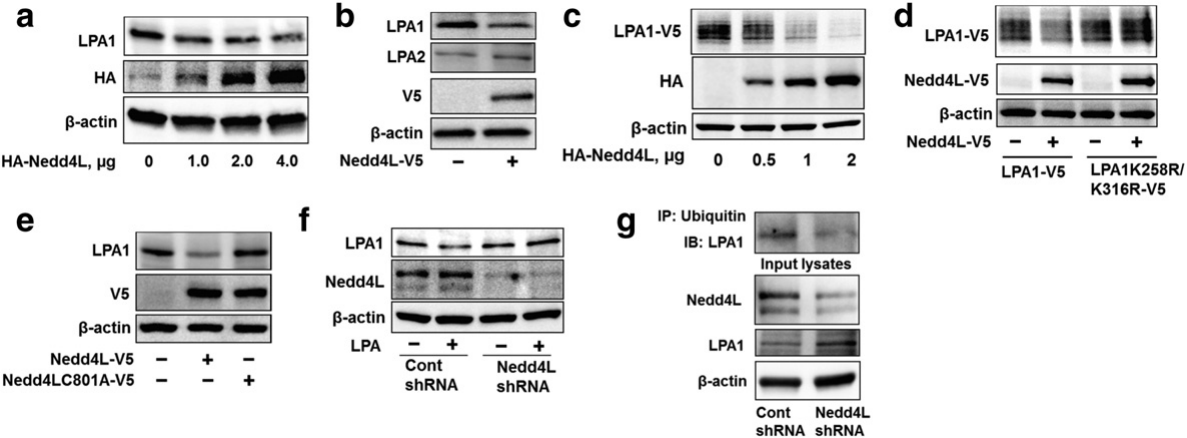
An E3 ubiquitin ligase, Nedd4L, induces LPA1 ubiquitination and degradation. **a**. MLE12 cells were transfected with HA-tagged Nedd4L (*HA-NEDD4L*, 0–4 μg) plasmid for 48 h. Cell lysates were analyzed by immunoblotting with LPA1, HA, and β-actin antibodies. **b**. HBEpCs were transfected with V5-tagged Nedd4L (*NEDD4L-V5*) plasmid for 48 h. Cell lysates were analyzed by immunoblotting with LPA1, LPA2, V5, and β-actin antibodies. **c**. MLE12 cells were co-transfected with *LPA1-V5* and *HA-NEDD4L* (0–2 μg) plasmids for 48 h. Cell lysates were analyzed by immunoblotting with V5, HA, and β-actin antibodies. **d**. MLE12 cells were co-transfected with *NEDD4L-V5* plasmid and *LPA1-V5* or *LPA1K258R&K316R-V5* plasmid for 48 h. Cell lysates were analyzed by immunoblotting with V5 and β-actin antibodies. **e**. MLE12 cells were transfected with *NEDD4L-V5* or *NEDD4LC801A-V5* plasmid for 48 h. Cell lysates were analyzed by immunoblotting with LPA1, V5, and β-actin antibodies. **f**. MLE12 cells were transfected with control shRNA (Cont shRNA) or *Nedd4l* shRNA for 72 h, and then cells were treated with LPA (5 μM) for 1 h. Cell lysates were analyzed by immunoblotting with LPA1, Nedd4L, and β-actin antibodies. **g**. MLE12 cells were transfected with Cont shRNA or *Nedd4l* shRNA for 72 h, and then cell lysates were subjected to immunoprecipitation with an ubiquitin antibody, followed by LPA1 immunoblotting. Cell lysates were analyzed by immunoblotting with LPA1, Nedd4L, and β-actin antibodies. Representative immunoblots were from at least three independent times.

Protein ubiquitination is mediated by a series of enzymatic reactions in which ubiquitin E3 ligase is responsible for transferring ubiquitin to the substrate. Over-expression of an E3 ubiquitin ligase, Nedd4L, reduced endogenous and over-expressed LPA1 levels in MLE12 (Fig. 2a, c) and human bronchial epithelial cells (HBEpCs) (Fig. 2b), without altering levels of LPA2 (Fig. 2b) and LPA1K258R&K316R (Fig. 2d). Inactive mutant of Nedd4L (Nedd4LC801A) had no effect on LPA1 degradation (Fig. 2e). Further, down-regulation of Nedd4L by *nedd4l* shRNA transfection attenuated LPA1 degradation (Fig. 2f) and ubiquitination (Fig. 2g). This study elucidates Nedd4L mediation of LPA1 site specific ubiquitination and degradation.

**Fig. 3:**
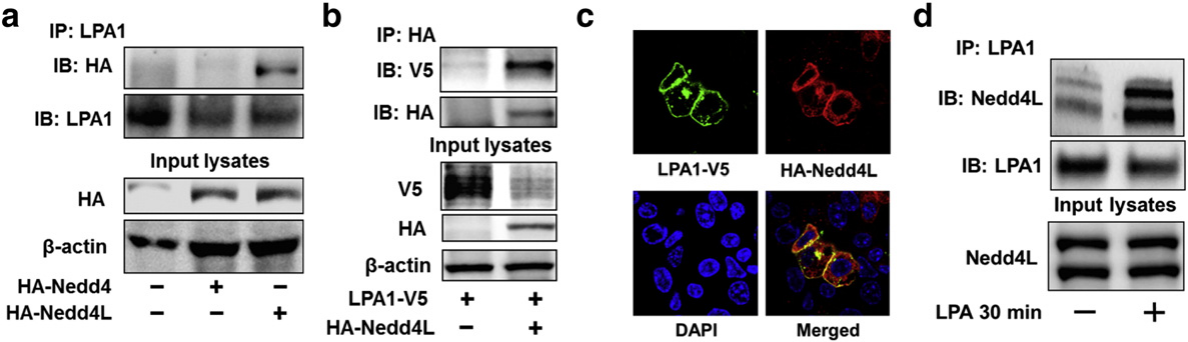
Nedd4L interacts with LPA1 on serine 319 residue. **a**. MLE12 cells were transfected with HA-Nedd4 or HA-Nedd4L plasmid for 48 h. Cell lysates were subjected to immunoprecipitation with a LPA1 antibody, followed by HA and LPA1 immunoblotting. Input lysates were analyzed by immunoblotting with HA and β-actin antibodies. **b**. MLE12 cells were transfected *LPA1-V5* with or without *HA-NEDD4L* plasmid. Cell lysates were subjected to immunoprecipitation with a HA antibody, followed by V5 and HA immunoblotting. Input lysates were analyzed by V5, HA, and β-actin antibodies. **c**. MLE12 cells grown on glass bottom dishes were co-transfected with *LPA1-V5* and *HA-NEDD4L* plasmid for 48 h, and then cells were fixed and immunostained with V5 and HA antibodies. LPA1-V5, green; HA-Nedd4L, red; nuclei, blue. **d**. MLE12 cells were treated with LPA (5 μM) for 30 min, and then cell lysates were subjected to immunoprecipitation with a LPA1 antibody, followed by Nedd4L and LPA1 immunoblotting. Input lysates were analyzed by Nedd4L immunoblotting. **e**. MLE12 cells were transfected with *LPA1-V5* or *LPA1S319A-V5* plasmid for 48 h. Cell lysates were subjected to immunoprecipitation with a V5 antibody, followed by Nedd4L immunoblotting. Input lysates were analyzed by immunoblotting with Nedd4L and β-actin antibodies. **f**. MLE12 cells were transfected with *LPA1-V5*, *LPA1S319A-V5*, or *LPA1S331A-V5*, with or without *HA-NEDD4L* plasmids for 48 h. Cell lysates were analyzed by V5, HA, and β-actin antibodies. Representative immunoblots were from at least three independent times.

To investigate whether Nedd4L interacts with LPA1, HA-Nedd4L or HA-Nedd4 (a Nedd4L isoform) over-expressing MLE12 cells were subjected to LPA1 immunoprecipitation, followed by HA immunoblotting. As shown in Fig. 3a, Nedd4L, not Nedd4, is associated with LPA1 (Fig. 3a). LPA1-V5 was also detectable in HA-Nedd4L immunoprecipated complex from LPA1-V5 and HA-Nedd4L co-over-expressing cells (Fig. 3b). Nedd4L and LPA1 were co-localized on the plasma membrane as well as in the cytoplasm (Fig. 3c). Further, we found that the association between LPA1 and Nedd4L was increased in response to LPA treatment (Fig. 3d). It is known that Nedd4L binds to proline-rich PY motif or serine residue of substrate; LPA1 has no canonical proline-rich PY motif, but a serine 319 mutant of LPA1 (LPA1S319A) is unable to interact with Nedd4L (Supplemental Fig. 3a), suggesting that serine 319 is a potential Nedd4L binding site within LPA1. LPA1S319A, but not other mutant (e.g. LPA1S331A), was resistant to LPA-induced receptor degradation (Supplemental Fig. 3b).

### 3.3 Nedd4L Inhibits the LPA-LPA1 Pathway

**Fig. 4:**
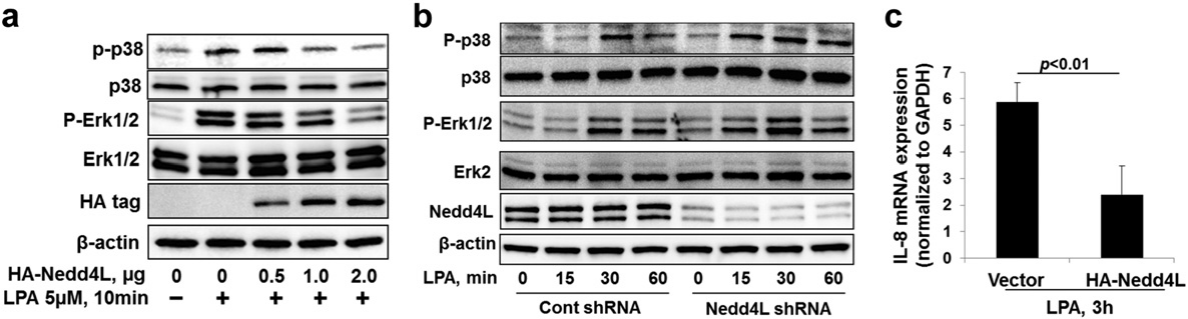
Nedd4L impairs LPA-LPA1 signaling. **a**. MLE12 cells were transfected with *HA-nedd4l* plasmids (0–2 μg) for 48 h, and then cells were treated with LPA (5 μM) for 10 min. Cell lysates were analyzed by immunoblotting with p-p38 MAPK, p38 MAPK, p-Erk1&2, Erk1&2, HA, and β-actin antibodies. **b**. MLE12 cells were transfected with Cont shRNA or *Nedd4l* shRNA for 72 h, and then cells were treated with LPA (5 μM) for 0–60 min. Cell lysates were analyzed by immunoblotting with p-p38 MAPK, p38 MAPK, p-Erk1&2, Erk1&2, Nedd4L, and β-actin antibodies. Representative immunoblots were from at least three independent times. **c**. HBEpCs were transfected with *HA-NEDD4L* plasmid for 48 h, and then cells were treated with LPA (1 μM) for 3 h. Cells were collected and IL-8 mRNA levels were analyzed by RT-realtime PCR.

To investigate the effect of Nedd4L on the LPA-LPA1 pathway, MLE12 cells were transfected with *HA-NEDD4L* plasmid or *Nedd4l* shRNA prior to LPA treatment. The phosphorylation of p38 MAPK and Erk1& 2 by LPA were attenuated in HA-Nedd4L over-expressing cells (Fig. 4a), while the phosphorylation was promoted in Nedd4L down-regulating cells (Fig. 4b). LPA is a strong stimulator of IL-8 mRNA expression in HBEpCs (Saatian et al., 2006). Further, we found that HA-Nedd4L attenuated LPA-induced IL-8 mRNA expression in HBEpCs (Fig. 4c). This study reveals that Nedd4L inhibits the LPA-LPA1 pathway through ubiquitination and reduction of LPA1 levels.

### 3.4 Deubiquitination of LPA1 by USP11 Increases its Stability

**Fig. 5:**
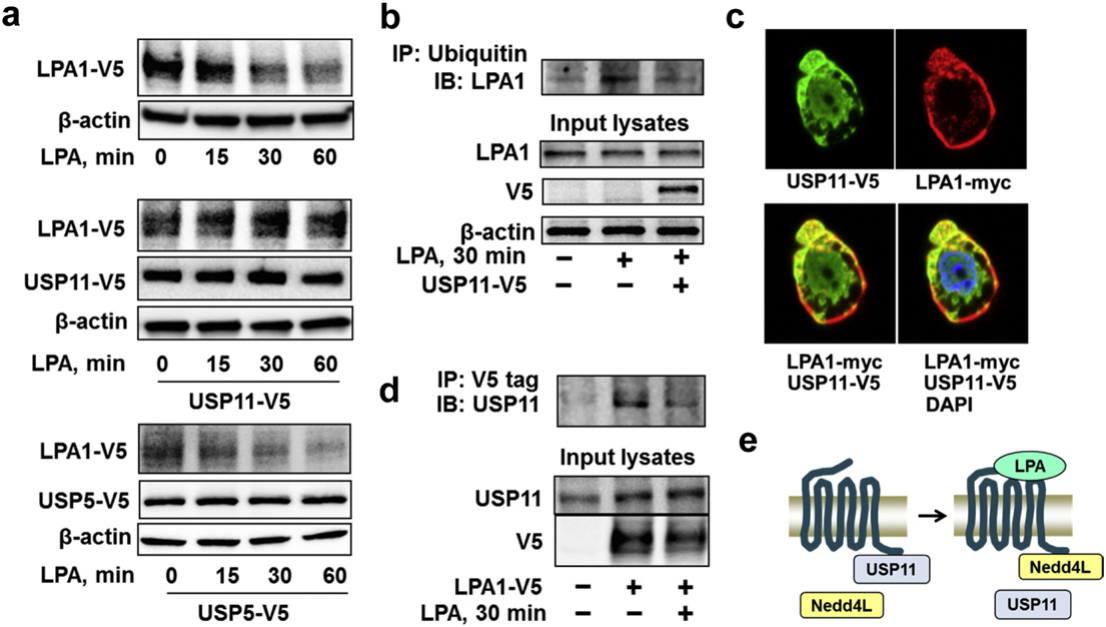
USP11 deubiquitinates and stabilizes LPA1. **a**. MLE12 cells were co-transfected with *LPA1-V5* and *USP11-V5* or *USP5-V5* plasmids for 48 h, and cells were treated with LPA (5 μM) for 0–60 min. Cell lysates were analyzed by immunoblotting with V5 and β-actin antibodies. **b**. MLE12 cells were transfected with USP11-V5 plasmid for 48 h, and then cells were treated with LPA (5 μM) for 30 min. Cell lysates were subjected to immunoprecipitation with an ubiquitin antibody, followed by LPA1 immunoblotting. Input lysates were analyzed by immunoblotting with LPA1, V5, and β-actin antibodies. **c**. MLE12 cells grown on glass bottom dishes were co-transfected with *Usp11-V5* and *Lpa1-myc* plasmids for 48 h. Cells were fixed and immunostained with V5 and myc antibodies. USP11-V5, green; LPA1-myc, red; nuclei, blue. **d**. MLE12 cells were transfected with *LPA1-V5* plasmid for 48 h, and then cells were treated with LPA (5 μM) for 30 min. Cell lysates were subjected to immunoprecipitation with a V5 antibody, followed by USP11 immunoblotting. Input lysates were analyzed by immunoblotting with USP11 and V5 antibodies. Representative immunoblots were from at least three independent times. **e.** Scheme shows that LPA treatment switches LPA1-USP11 complex to LPA1-Nedd4L complex.

Ubiquitination is reversible and deubiquitination is catalyzed by a family of deubiquitinating enzymes. We found that over-expression of USP11, but not other deubiquitinating enzyme such as USP5, attenuated LPA-induced LPA1-V5 degradation (Fig. 5a). Over-expression of USP11 reversed LPA-induced LPA1 ubiquitination (Fig. 5b), suggesting that USP11 stabilizes LPA1 through deubiquitination of the receptor. Co-immunofluorescence staining revealed that USP11 was co-localized with LPA1 in both the plasma membrane and cytoplasm (Fig. 5c); this association was also confirmed by immunoprecipitation (Fig. 5d). In contrast to the LPA-increased in the association between LPA1 and Nedd4L, LPA treatment reduced the interaction between LPA1 and USP11 (Fig. 5d). Taken together, USP11 deubiquitinates and stabilizes LPA1, and LPA-induced switching LPA1 association with USP11 to Nedd4L plays a critical role in LPA1 ubiquitination and degradation (Fig. 5e).

### 3.5 USP11 Promotes the LPA-LPA1 Signal Pathway

**Fig. 6:**
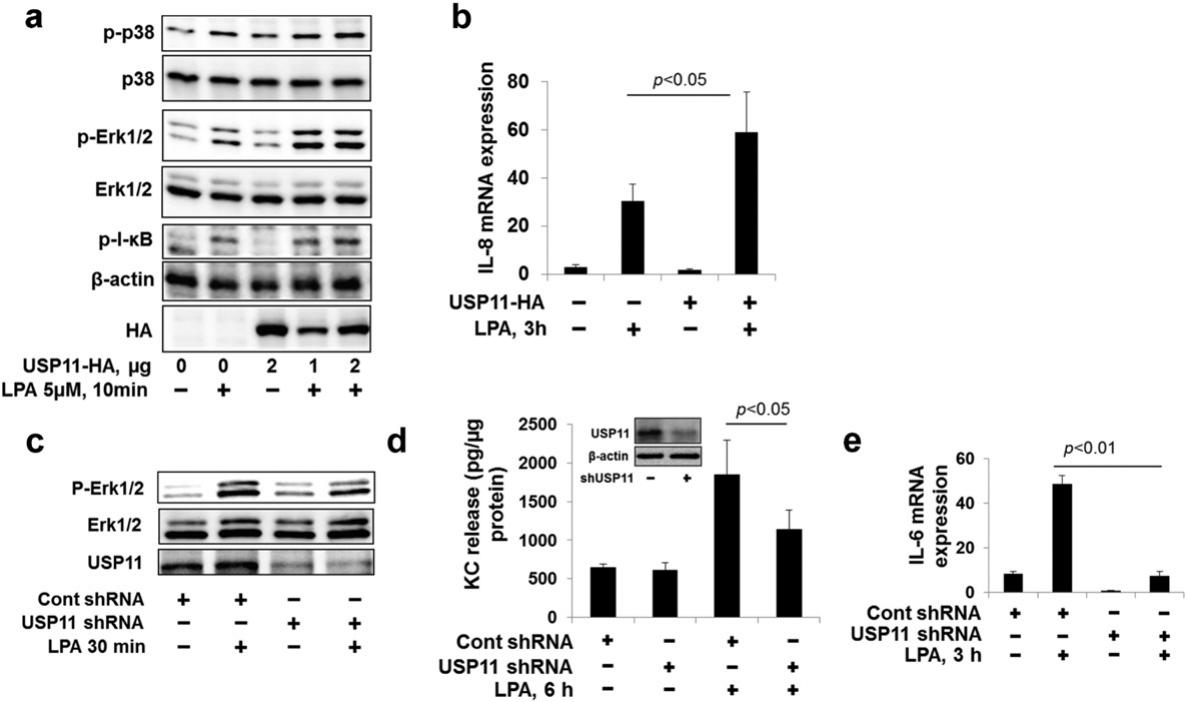
USP11 promotes LPA-LPA1 signaling. **a**. MLE12 cells were transfected with *USP11-HA* plasmid (0–2 μg) for 48 h, and then cells were treated with LPA (5 μM) for 10 min. Cell lysates were analyzed by immunoblotting with p-p38 MAPK, p38 MAPK, p-Erk1&2, Erk1&2, p-IκB, HA, and β-actin antibodies. Representative immunoblots were from at least three independent times. **b**. HBEpCs were transfected with *USP11-HA* plasmid for 48 h, and then cells were treated with LPA (1 μM) for 3 h. Cells were collected and IL-8 mRNA levels were examined by RT-realtime PCR. **c**. MLE12 cells were transfected with *Usp11* shRNA for 72 h, and then cells were treated with LPA (5 μM) for 10 min. Cell lysates were analyzed by immunoblotting with p-Erk1&2, Erk1&2, and HA antibodies. Representative immunoblots were from at least three independent times. **d**. MLE12 cells were transfected with *usp11* shRNA for 72 h, and then cells were treated with LPA (5 μM) for 6 h. Cell culture supernatants were collected and KC levels in the media were analyzed by Elisa. The expression of USP11 was determined by immunoblotting with USP11 and β-actin antibodies (insert). **e**. HBEpCs were transfected with *USP11* shRNA for 72 h, and then cells were treated with LPA (1 μM) for 3 h. Cells were collected and IL-6 mRNA levels were analyzed by RT realtime PCR.

To investigate whether USP11 affects the LPA-LPA1 signal pathway, MLE12 cells or HBEpCs were transfected with *USP11-HA* or *Usp11 shRNA* plasmid prior to LPA treatment. LPA-induced phosphorylation of p38 MAPK, Erk1&2, and I-κB were increased in USP11-HA over-expressing MLE12 cells (Fig. 6a). Over-expression of USP11-HA enhanced LPA-induced IL-8 gene expression in HBEpCs (Fig. 6b). Down-regulation of USP11 attenuated LPA-induced phosphorylation of Erk1& 2, KC release, and IL-6 gene expression in MLE12 cells (Fig. 6c-6e). The data indicates that USP11 promotes LPA-LPA1 signaling and LPA-mediated cytokine release by deubiquitinating and stabilizing LPA1.

### 3.6 USP11 Modulates the LPS Pathway by Increasing LPA1-CD14 Complex Levels

**Fig. 7:**
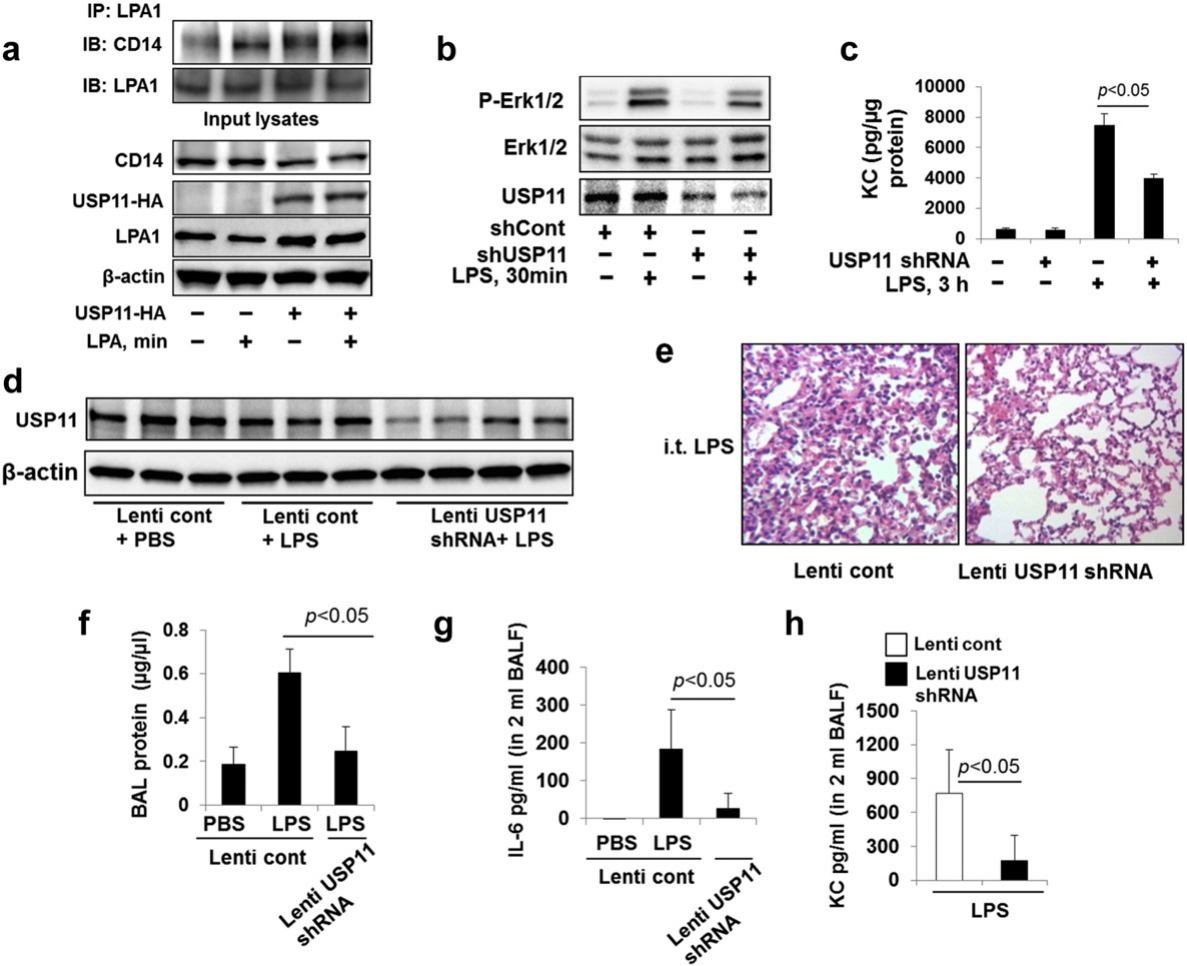
USP11 promotes LPA1-CD14 interaction and LPS-induced lung inflammation. **a**. MLE12 cells were transfected with *USP11-HA* plasmid for 48 h, and then cells were treated with LPA (5 μM) for 30 min. Cell lysates were subjected to immunoprecipitation with LPA1 antibody, followed by CD14 and LPA1 immunoblotting. Input lysates were analyzed by immunoblotting with CD14, HA, LPA1, and β-actin antibodies. **b**. MLE12 cells were transfected with *usp11* shRNA for 72 h, and then cells were treated with LPS (10 μg/ml) for 30 min. Cell lysates were analyzed by immunoblotting with p-Erk1&2, Erk1&2, and USP11 antibodies. Representative immunoblots were from at least three independent times. **c**. MLE12 cells were transfected with *Usp11* shRNA for 72 h, and then cells were treated with LPS (10 μg/ml) for 3 h. Cell culture supernatants were collected and KC levels in the media were analyzed by Elisa. **d**–**h**. C57/BL6 mice (6–8/group) were intratracheally infected with control lentivirus (Lenti cont, 1 × 10^9^ cfu/mice) or lentiviral vector carrying *usp11* shRNA (Lenti USP11 shRNA Lenti cont, 1 × 10^9^ cfu/mice) for 7 days, followed by i.t. administration with LPS (2 mg/kg body weight) for 24 h. Lung tissue lysates were analyzed by immunoblotting with USP11 and β-actin antibodies (**d**). Lung tissue were fixed and stained with H&E (**e**). BAL was collected, and then protein levels were examined by protein assay (**f**), IL-6 (**g**), and KC (**h**) were examined by Elisa.

It has been shown that LPA1 interacts with CD14, a LPS co-receptor, in response to LPS treatment (Zhao et al., 2011). Inhibition or down-regulation of LPA1 attenuated LPS-induced signaling and cytokine release in lung epithelial cells (Zhao et al., 2011, He et al., 2009). Here we found that LPA treatment also induced the association between LPA1 and CD14 (Fig. 7a), which was promoted by over-expression of USP11-HA. This suggests that USP11 may modulate the LPS pathway through regulating LPA1 stability and levels of LPA1-CD14 complex. To investigate whether USP11 modulates LPS-induced signaling, USP11 expression was down-regulated by *usp11* shRNA transfection. Down-regulation of USP11 attenuated LPS-induced phosphorylation of Erk1& 2 (Fig. 7b) and cytokine KC release (Fig. 7c) in MLE12 cells. It has been shown that LPA1 deficient mice exhibited significant reduction of LPS-induced inflammatory responses, compared to wild type mice, in a murine model of lung injury (Zhao et al., 2011). To investigate whether down-regulation of USP11 exhibits protective effects against LPS-induced lung injury, USP11 levels in the mouse lungs were down-regulated by *usp11* shRNA in a lentiviral vector delivery system (Fig. 7d). Down-regulation of USP11 diminished LPS-induced neutrophil influx, BAL protein, IL-6 and KC levels (Fig. 7e-7h). This data provides a potential target in lessening inflammatory lung injury, and builds a molecular basis for development of a therapeutic strategy to diminish endotoxin-induced pro-inflammatory responses through inhibition of USP11.

### 3.7 USP11 Inhibitor, Mitoxathrone, Reduces LPA1 Stability and Protects LPS-Induced Lung Injury.

**Fig. 8:**
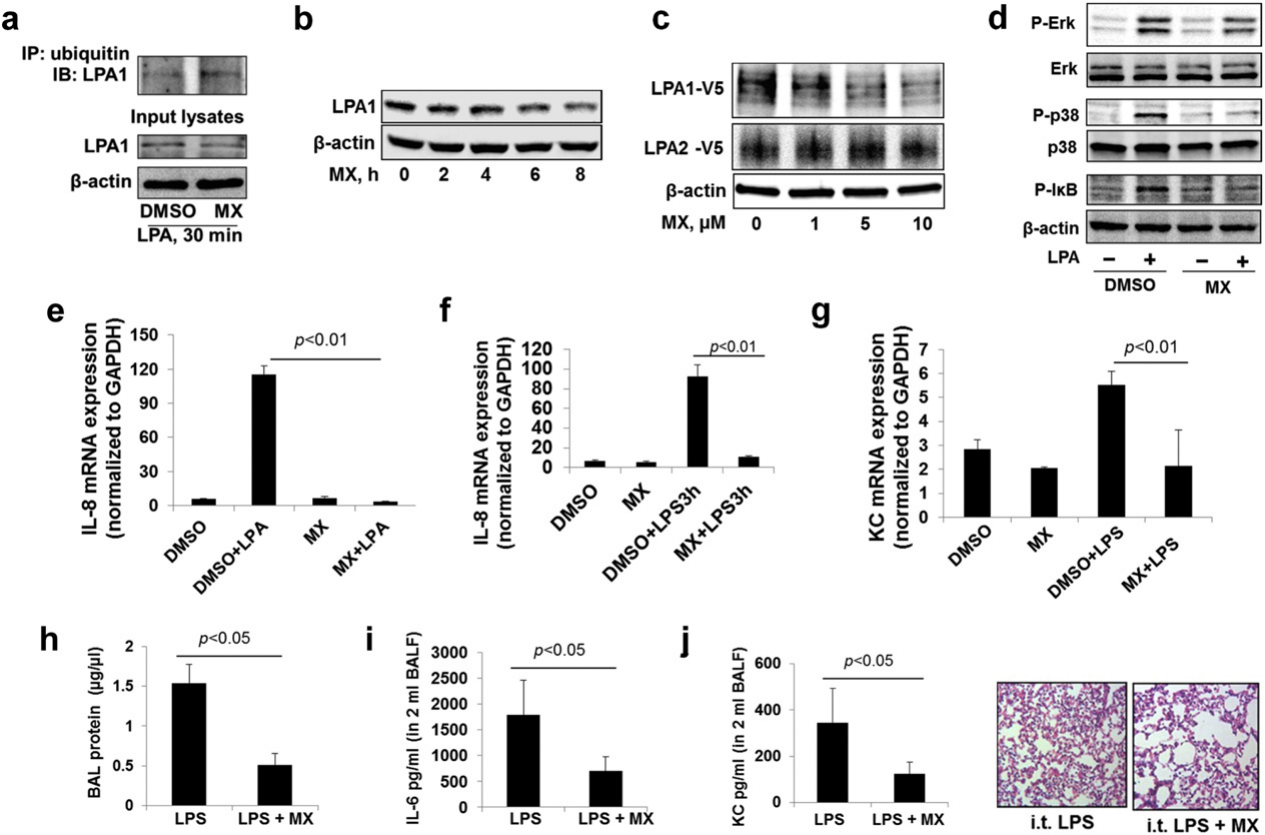
MX, an inhibitor of USP11, reduces LPA1 levels and lessens LPS-induced lung inflammation. **a**. MLE12 cells were treated with DMSO or MX (5 μM) for 1 h prior to LPA treatment (5 μM, 30 min). Cell lysates were subjected to immunoprecipitation with an ubiquitin antibody, followed by LPA1 immunoblotting. Input lysates were analyzed by immunoblotting with LPA1 and β-actin antibodies. **b**. MLE12 cells were treated with MX (5 μM) for 0–8 h. Cell lysates were analyzed by immunoblotting with LPA1 and β-actin antibodies. **c**. MLE12 cells were transfected with *LPA1-V5* or *LPA2-V5* plasmid, and then cells were treated with MX (0–10 μM) for 6 h. Cell lysates were analyzed by immunoblotting with antibodies to V5 and β-actin antibodies. **d**. MLE12 cells were treated with MX (5 μM, 6 h) prior to LPA treatment (5 μM, 10 min). Cell lysates were analyzed by p-Erk1&2, Erk1&2, p-p38 MAPK, p38 MAPK, p-I-κB, and β-actin antibodies. Representative immunoblots were from at least three independent times. **e**. HBEpCs were treated with MX (5 μM, 6 h) prior to LPA treatment (1 μM, 3 h), and then cells were collected and IL-8 mRNA levels were examined by RT realtime PCR. **f**. HBEpCs were treated with MX (5 μM, 6 h) prior to LPS treatment (10 μg/ml, 3 h), and then cells were collected and IL-8 mRNA levels were examined by RT realtime PCR. **g**. MLE12 cells were treated with MX (5 μM, 6 h) prior to LPS treatment (10 μg/ml, 6 h), and then cells were collected and KC mRNA levels were examined by RT realtime PCR. **h**–**k**. C57/BL6 mice (6–8/group) were i.t. administrated with MX (0.25 mg/kg body weight) for 1 h, and then followed by i.t. LPS (2 mg/kg body weight) for 24 h. BAL were collected and BAL protein levels were measured by protein assay (**h**), IL-6 (**i**) and KC (**j**) levels were examined by Elisa. Lung tissues were fixed and stained with H&E (**k**).

Mitoxantrone (MX), an anti-cancer drug, was reported to inhibit USP11 (Burkhart et al., 2013). To confirm the data from using *usp11 shRNA*, mitoxantrone was used to examine the role of USP11 in regulation of LPA1 stability, the LPA-LPA1 pathway, and LPS signaling. MX increased LPA-induced ubiquitination of LPA1 (Fig. 8a), and reduced the mass of endogenous (Fig. 8b) and over-expressed LPA1 (Fig. 8c), without altering LPA1 mRNA levels in MLE12 (Supplementary Fig. 4). Further, MX pretreatment blocked LPA-induced phosphorylation of Erk1& 2, p38 MAPK, and I-κB (Fig. 8d), IL-8 gene expression, as well as LPS-induced IL-8 release in HBEpCs, and KC gene expression in MLE12 (Fig. 8e–8g). To investigate whether MX exhibits anti-inflammatory effects in LPS-induced lung injury, MX was intratracheally administrated into mouse lungs prior to intratracheal LPS challenge. MX treatment significantly attenuated LPS-induced increases in levels of BAL protein, IL-6, and KC, and neutrophil influx into alveolar spaces (Fig. 8h–8k). This data indicates that down-regulation or inhibition of USP11 protects against lung inflammation via reduction of LPA1 stability and attenuation of the LPA1-CD14 pathway.

## 4 Discussion

GPCR is a large family of cell surface receptors, which contributes to the pathogenesis of a variety of inflammatory diseases, and is widely targeted in drug discovery. As most GPCRs are ubiquitously expressed in human cells, antagonists of GPCRs may cause unexpected side effects due to complete inhibition of the GPCR pathway (Giulio Innamorati et al., 2011). Thus, there is an unmet need to test an advanced strategy that will suppress GPCR protein stability with limited off-target effects, without completely inhibiting the target. Ubiquitination of cell surface receptors regulates their stability, thus mediating downstream signaling of the receptors. LPA1 is a well characterized GPCR as its expression and it-mediated pathways are related to the pathogenesis inflammatory lung diseases and tumors (Tager et al., 2008, Saatian et al., 2006, Zhao et al., 2011, Lee et al., 2015), however, its stability has not been investigated. The current study reveals that LPA1 ubiquitination and degradation is mediated by E3 ubiquitin ligase Nedd4L, which is reversed by deubiquitinating enzyme USP11. This study reveals that upon stimulation by an agonist, a GPCR switches its association with the deubiquitinating enzyme (stabilizer) to the ubiquitin E3 ligase (degrader), causing its ubiquitination and degradation. The discovery of the molecular regulation of LPA1 stability will further lead to development of a unique strategy to attenuation of LPA1 pathway through inhibition of USP11.

LPA1 is ubiquitously expressed in mammalian cells, and it contains an extracellular domain, three extracellular loops, seven transmembrane domains, three intracellular loops, and C-terminal tail (Chrencik et al., 2015, Murph et al., 2008). Recent studies have revealed that intracellular trafficking of LPA1 determines the levels of LPA1 on the cell surface (Murph et al., 2008, Zhao et al., 2014). β-arrestin- and clathrin-dependent endocytosis mediate LPA1 internalization(Urs et al., 2005), while heat shock protein regulates LPA1 trafficking from the endoplasmic reticulum to the plasma membrane (Zhao et al., 2014, Dong et al., 2007). In addition to intracellular trafficking, the receptor stability on the cell surface determines receptor-mediated biological functions. Reduction of stability of sphingosine-1-phopshoate receptor 1 (S1P1), which belongs to the same superfamily as LPA1, leads to endothelial barrier dysfunction (Oo et al., 2011). The current data indicates that LPA1 is a substrate of Nedd4L, and that Nedd4L diminishes LPA1 stability, as well as LPA-induced signals and cytokine release. LPA1 is mono-ubiquitinated at lysine 258 and 316, which are localized in the third intercellular loop and C-terminal tail, separately. This data is consistent with the emerging evidence showing that mono-ubiquitination of membrane receptors leads to receptor lysosomal degradation. Though several studies indicate that mono-ubiquitination is an internalization signal for membrane proteins, such as α-factor receptor (Lucero et al., 2000, Terrell et al., 1998), however, LPA-induced LPA1 internalization is not dependent on its ubiquitination at K258 and K316 residues, as a mutant with both lysine residues (LPA1K258R&K316R) internalizes the same as LPA1 wild type in the setting of LPA treatment. Similarly, the ubiquitination of S1P1 regulates its degradation without affecting its internalization, suggesting that ubiquitination is not essential for GPCR internalization (Oo et al., 2007). Taken together, LPA1 is ubiquitinated in response to agonist ligation, which is catalyzed by Nedd4L E3 ligase. The ubiquitination of LPA1 causes its lysosomal degradation and limits LPA1-mediated cytokine release. It is known that Nedd4L contributes to the pathogenesis of lung inflammatory diseases (Kimura et al., 2011, Boase et al., 2011). Nedd4L knockout mice exhibit respiratory distress and cystic fibrosis-like disease (Boase et al., 2011, Rotin and Staub, 2012). Most studies have been focusing on Nedd4L regulation of ENaC stability (Kimura et al., 2011, Rotin and Staub, 2012). Recent studies suggest that LPA1 plays a critical role in the pathogenesis of pulmonary fibrosis, as knockdown or inhibition of LPA1 lessens progress of pulmonary fibrosis (Swaney et al., 2010, Castelino et al., 2011). This study provides evidence that Nedd4L regulates LPA1 stability, suggesting that the anti-fibrotic effect of Nedd4L is through targeting both ENaC and LPA1 for regulating their stability. The effect of Nedd4L on lung injury has been revealed (Boase et al., 2011). Here, we reveal that Nedd4L attenuates LPA-induced IL-8 release, suggesting Nedd4L may have an anti-inflammatory property through regulating LPA1 signaling.

Most studies have been focused on investigating the molecular control of GPCR ubiquitination by identifying the E3 ubiquitin ligase, while the little information is available for deubiquitination of GPCR by deubiqutinating enzymes. Here we report that a deubiquitinating enzyme, USP11, promotes LPA1 stability by reduction of LPA1 ubiquitination, resulting in enhanced LPA-LPA1 signal pathway. USP11 has been known to regulate stability of ALK5 (Al-Salihi et al., 2012) and promyelocytic leukemia protein (Wu et al., 2014). This report reveals a role of USP11 in the regulation of GPCR stability. An important discovery in this study is that a change of association of LPA1 with USP11 to Nedd4L is triggered by ligand binding. The results provide a model for ubiquitination related enzymes regulation of substrate stability by switching their interaction with substrate. It is this switch between deubiquitination enzyme-GPCR to E3 ubiquitin ligase-GPCR which determines the degree of ubiquitination and degradation of GPCR. The serine 319 was identified as the Nedd4L binding site in LPA1, while the USP11 binding site in LPA1 and how ligand treatment shifts the LPA1 binding from USP11 to Nedd4L are still unclear. It is possible that ligand-induced receptor conformational change triggers the receptor binding from its stabilizer (deubiquitinating enzyme) to destabilizer (ubiquitin E3 ligase). USP11 also deubiquitinates SUMO-ubiquitin chains from PML (Wu et al., 2014), while the sumolytion of LPA1 has not been discovered.

LPA1 is recognized as pro-inflammatory GPCR in the lung inflammatory diseases (Tager et al., 2008, Zhao et al., 2011, Zhao et al., 2006). LPA induced cytokine release in lung epithelial cells (Cummings et al., 2004, Zhao et al., 2005, Saatian et al., 2006), leading to recruit neutrophil influx (He et al., 2009). Recent studies have shown that LPA1 interacts with CD14, co-receptor of LPS (Zhao et al., 2011, Zhao et al., 2015). Understanding regulation of LPA1 stability may provide an advanced therapeutic strategy to lessen inflammatory lung diseases by down-regulating, but not completely inhibiting, LPA1 levels. The current study shows that USP11 has pro-inflammatory effect in lung inflammatory injury. This is contrast with the previous finding that USP11 negatively regulates TNFα-induced cytokine release by targeting NF-κB pathway in Hela cells (Sun et al., 2010). The controversial conclusion may be due to using different cell types and stimuli. This study shows that USP11 stabilizes LPA1, thus leading an increase in LPA1-CD14 complex on the cell surface, which contributes LPS-induced signaling and cytokine release in lung epithelial cells. This finding indicates that targeting USP11 attenuates endotoxin-induced inflammatory responses in the murine model of lung injury.

This study reveals molecular mechanisms by which Nedd4L and USP11 regulate LPA1 stability and LPA1-mediated signaling, thus modulate LPA- or LPS-induced inflammatory responses. Destabilization of LPA1 by inhibition or down-regulation of USP11 diminishes lung injury. The anti-inflammatory effect of USP11 inhibitor may be beneficial to other diseases including inflammatory bowel disease. To develop small molecules specific for destabilization of LPA1, future studies will focus on drug throughput screening of small molecules to interrupt the interaction between LPA1 and USP11 interaction, thereby leading destabilization of LPA1 and lessening endotoxin-induced inflammatory lung injury.

## Conflict of Interest Statement

No conflicts to report.

## Author Contributions

J.Z. and Y.Z. jointly designed, performed experiments, analyzed the data and wrote the manuscript; J.W., S.D., R.K.M., A.M.J., and L.Z. performed experiments and analyzed the data; Y.Z. oversaw and directed the study.

## Appendix A Supplementary data

Supplementary figures. Image 1
