# Supplementary material for: Destabilization of Lysophosphatidic Acid Receptor 1 Reduces Cytokine Release and Protects Against Lung Injury
Source: eBioMedicine. 2016 Jul 18;10:195–203. doi: 10.1016/j.ebiom.2016.07.020 (PMC5006730; doi:10.1016/j.ebiom.2016.07.020)
Supplement: Supplementary file 1 — Supplementary figures. [file mmc1.docx]

Supplmentary materials

**Destabilization of lysophosphatidic acid receptor 1 reduces cytokine release and protects against lung injury**

Jing Zhao ^1^, Jianxin Wei ^1^, Su Dong ^1, 2^, Rachel K Bowser ^1^, Lina Zhang ^3^, Anastasia M Jacko ^1^, Yutong Zhao ^1#^

From the ^1^ Department of Medicine, University of Pittsburgh School of Medicine, Acute Lung Injury Center of Excellence, Vascular Medical Institute, and Department of Cell Biology, University of Pittsburgh, Pittsburgh, PA, United States; ^2^ Department of Anesthesia, First Hospital of Jilin University, Changchun, China. ^3^ Department of Critical Care Medicine, Xiangya Hospital, Central South University, Changsha, Hunan, China.

**
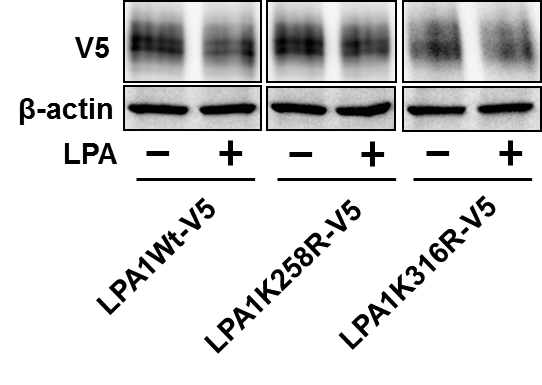
**

**Supplementary Figure 1. LPA induces degradation of single lysine mutants of LPA1.** MLE12 cells were transfected with *LPA1-V5*, *LPA1K258R-V5*, or *LPA1K316R-V5* plasmid for 48 h, and then cells were treated with LPA (5 µM) for 1 h. Cell lysates were analyzed by immunoblotting with V5 and β-actin antibodies. Representative immunoblots were from at least three independent times.


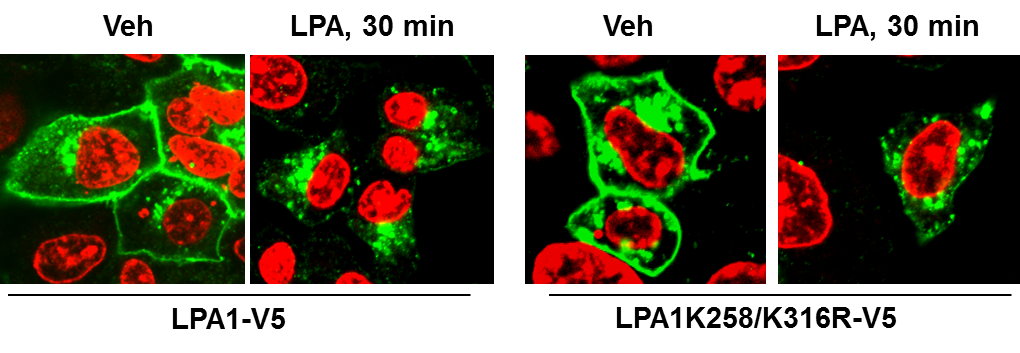


**Supplementary Figure 2. LPA induces both LPA1 wild type and LPA1K258R/K316R internalization.** MLE12 cells grown on glass bottom dishes were transfected with *LPA1-V5* or *LPA1K258R&K316R-V5* plasmid for 48 h, and then cells were fixed and immunostained with a V5 antibody. LPA1-V5 and LPA1K258R&K316R-V5, green; nuclei, red. Representative images were from at least three independent times.


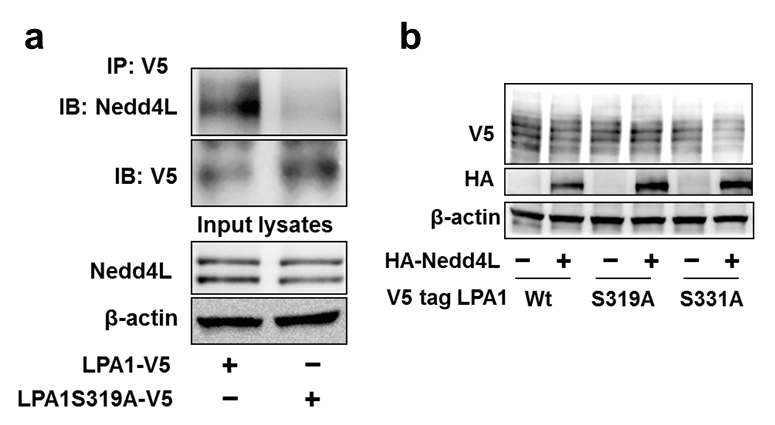


**Supplementary Figure 3. Serine 319 is the binding site for Nedd4L. a.** MLE12 cells were transfected with *LPA1-V5* or *LPA1S319A-V5* plasmid for 48 h. Cell lysates were subjected to immunoprecipitation with a V5 antibody, followed by Nedd4L and V5 immunoblotting. Input lysates were analyzed by immunoblotting with Nedd4L and β-actin antibodies. **b.** MLE12 cells were transfected with *LPA1-V5*, *LPA1S319A-V5*, or *LPA1S331A-V5*, with or without *HA-NEDD4L* plasmids for 48 h. Cell lysates were analyzed by V5, HA, and β-actin antibodies. Representative immunoblots were from at least three independent times.


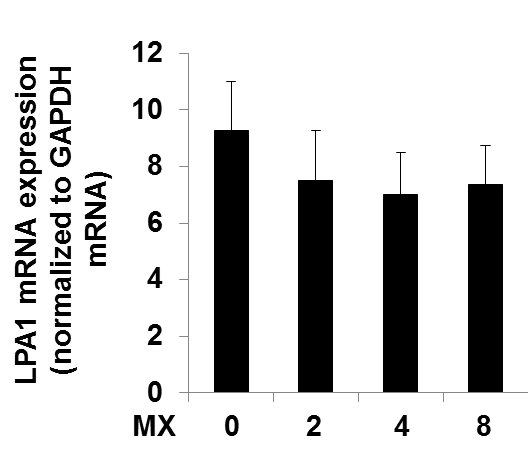


**Supplementary Figure 4. MX has no effect on LPA1 mRNA expression.** MLE12 cells were treated with MX (10 µM) for 0-8 h, and then total RNA was extracted. LPA1 mRNA levels were examined by RT-realtime PCR.
